# Is Silver the Ultimate Antimicrobial Bullet?

**DOI:** 10.3390/antibiotics7040112

**Published:** 2018-12-19

**Authors:** Raymond J Turner

**Affiliations:** Department of Biological Sciences, University of Calgary, Calgary, Alberta T2N 1N4, Canada; turnerr@ucalgary.ca; Tel.: +1-403-220-4308

The use of metal compounds as antimicrobial agents has been around since antiquity, only to be replaced by the introduction of organic antibiotics and antiseptics in the mid-20th century. The discovery of penicillin by Alexander Fleming in 1928 began the era of antibiotics. Unfortunately, this time is rapidly coming to an end, as antibiotic resistance is now the norm for most pathogen strains. We now accept that we have entered the Antibiotic Resistance Era, where the World Health Organization considers antibiotic resistance one of the biggest threats to global health, food security and development today. Their 2017 report confirms the world is running out of useful antibiotics [1]. Since the turn of the century, interest into alternatives to antibiotics has seen an explosion of attention into inorganic antimicrobial agents including metal-based antimicrobials [2].

Beyond its malleable and aesthetic qualities, silver has been used since antiquity to control infection. For example, ancient mariners would toss silver coins into the drinking water barrel on ships to prevent fouling. Nowadays, silver and silver nanoparticles (AgNPs) are widely used in healthcare, food industry, cosmetic industry, coatings to surface materials and in textiles. Most of these applications are targeting for infection control or treatment, however, in textiles the antimicrobial properties are exploited for odor control.

Considerable efforts have been made towards understanding the molecular mechanism(s) of action of silver [3,4]. The rules for efficacy of metals as antimicrobials are poorly understood but may follow some fundamental chemical rules (discussed in [5]). In the case of silver, there are likely multiple targets, both direct and indirect, leading various cellular systems to be affected [6]. Regardless of the efficacy, bacteria can develop resistance to metal-based antimicrobials [7], with a silver resistance determinant identified as early as 1975, primarily through an efflux mechanism as well as others (reviewed in [8]).

With a few exceptions, the articles of this special issue of ‘Silver-based antimicrobials’ focus on AgNPs or nanomaterials, which reflects field-wide research trends. Different AgNP synthesis methods or formulations that are in combination with other antimicrobials are of interest. The various methods, either biological or chemical-physical, produce different types of AgNPs. The articles here and the review of patents from Sim et al., [9] reflects an explosion of such exploratory activity towards potential industrial and health care applications. It is becoming clear that the different formulations of AgNPs that lead to differences in their size, shape, structure and their release of Ag atoms lead to very different antimicrobial activities. This body of work suggests the possibility of tuning silver’s antimicrobial activity towards specific strains. Research to date suggests AgNPs to be very effective antimicrobial silver bullets.

As we research the mechanisms of toxicity and resistance of silver, as well as how to prepare novel AgNP formulations, we must keep in mind how we intend to use silver in order to preserve its efficacy. It is prudent to consider stewardship and sustainability at the start before misuse runs rampant. However, given the present overuse of silver in textiles, are we already too late?
